# Intellectual Property Management Capacity in Tanzania: Perception of Researchers in Academia and Research Institutions of Health and Allied Sciences

**DOI:** 10.24248/eahrj.v6i2.697

**Published:** 2022-11-30

**Authors:** Kijakazi Obed Mashoto

**Affiliations:** National Institute for Medical Research

## Abstract

**Background:**

Intellectual Property (IP) management is a fundamental element in putting intellectual property to work for the public good. This study aimed at assessing the perception of the research community on intellectual Property Management (IPM) capacity in universities of health and allied sciences, and health research institutions in Tanzania.

**Methods:**

A total of 148 respondents which included scientists, researchers and postgraduate students from 18 institutions in Tanzania returned the filled in self-administered online questionnaire (59.4% response rate).

**Results:**

Most respondents (76.5%) were of the view that social and economic development are the priorities of their institutions but not intellectual property (IP) commercialisation as only a few (18%) reported that their institutions have arrangement with local industries and 22% said that their institutions have functioning intellectual Property Management Office (IPMO). About 30% of the respondents reported that IP policies exist in their institutions. In most cases, respondents were of the view that the need for effective management of IP (86.7%) triggered the institution's decision to have IP policy. Among the respondents who stated that their institutions have IP policy, slightly over one third to a half acknowledged that their institutions' IP policies intend to regulate mechanisms for benefit sharing and IP ownership.

Among those who reported that their institutions had IP policies, only 12.8% indicated that the policies were being implemented. Majority of respondents (80.4%) knew the existence of employment contracts but, only 28.4% signed the contract and 12.8% were well informed that they had been employed to invent. Over 20% of respondents said that their institutions had the capacity to exploit and manage IP and only a quarter of respondents reported to have capacity for IP management. Less than 40% of respondents admitted that their institutions had entrepreneurship capacity and 30% affirmed that their institutions were capable of establishing IPMO.

**Conclusion:**

Opinions of the respondents indicate that universities and health research institutions in Tanzania have inadequate capacity for IPM due to inadequate or lack of frameworks, mechanisms, structures and resources for protection of generated IP. Technical and financial support are needed to strengthen capacity for IPM in universities and health research institutions in Tanzania.

## BACKGROUND

The importance of research undertaken within universities and research institutions is widely recognized by governments, industries and diverse stakeholders. However, in Tanzania, the contribution of universities and research institutions in the generation of new ideas and knowledge as an economic driver, has never been higher.^1–3^At the same time, universities are faced with a rapidly changing environment shaped by pressure on funding, an emphasis on quality assurance and the increasing impact of globalization, marketization and new technology.^4^Such pressures for change have placed a particular emphasis on the need for effective intellectual property management (IPM) in higher learning and research institutions.^5–9^Intellectual property rights (IPRs) play an essential role in the safety and protection of the knowledge produced and thus IPM is a fundamental element in putting intellectual property (IP) to work for the public good. The IP strategy consists of a set of measures, formulated and implemented by an institution. These measures encourage and facilitate the effective creation, development, management, and protection of IP^10–11^.

Industrial context and institutional setting matter tremendously when it comes to how IP is constructed, used and deployed. In other words, the impact of IP depends on how it is used, who uses it, and for what purpose. The rewards from successful IPM can be enormous, but without effective IPM skills, universities and research institutions risk squandering the rights, powers, and opportunities that the IP system provides. Thus, it is important to invest in the tools, people and processes in order to improve and maximise IPM, revenue generation activities and increase the IP value within the organisation.^12^

The IP strategies can serve to either restrict or expand access to innovations, research results and data. Failure of the organization to obtain and maintain rights for the generated IP may result in other entities appropriating elements of the value without major regard to the mission of institution in question, or it could lead to the intellectual assets becoming useless due to lack of further investment and development.^13^ The aim of this study was to assess research communities' perception on the capacity for IPM in academia and research institutions of health and allied sciences in Tanzania.

## METHODS

### Study Population and Sample Size

Study population included researchers, scientists, academicians and post graduate students from health and allied science universities and research institutions in Tanzania. With the population of 3,083 researchers and scientists in the targeted health and allied sciences institutions^14–15^ and assumption that 50% of targeted population perceive that universities and research institutions have capacity for IPM, provided that the level of confidence is 90% and accepted margin of error is 5%, the calculated sample size was 249.^16^

### Sampling Procedures

Three research institutions (1 public and 2 private), 6 out of 12 public universities and 4 out of 18 private universities were purposively selected because they are institutions of health and allied sciences. In each selected university and research institute, all researchers including postgraduate students were eligible for the survey, and hence administrators of the selected institutions were asked to share the survey link to their researchers and postgraduate students through email, WhatsApp and Twitter. Potential study participants were requested to respond to the questionnaire within the allocated time. Therefore, study participants were self-selected.

### Study Design

A cross section survey was conducted to assess IP capacity in health research institutions and universities in Tanzania. All researchers, scientists, academia and postgraduate students from health research institutions and universities in Tanzania were eligible to participate in this study.

### Data Collection

Administrators of the selected health and allied sciences institutions were asked to share the questionnaire link with their researchers and postgraduate students. The targeted health and allied sciences institutions included 3 research institutions (1 public and 2 private), 6 out of 12 public universities and 4 out of 18 private universities. The online self-administered questionnaire was distributed to all researchers and postgraduate students in the targeted academic and research communities in Tanzania through emails, WhatsApp and Twitter. However, the link was active from the 2^nd^ to the 4^th^ week of May 2021. Thus, analysis was based on individuals who responded to the questionnaire within the allocated time. Data collection tool was designed to collect information on IP existence of IP policy, agreements and guidelines, reasons for developing IP policy and implementation status, institutions' commercialization strategies, types of commercialized and granted IPRs, institutions' capacity and individuals' skills and knowledge for IPM, and entrepreneurial environments and capacity. For each question, respondent was required to select one of the three pre-determined responses (agreed, disagreed or not sure).

### Data Analysis

Descriptive and cross tabulation analysis were conducted using IBM SPSS Statistics for Windows version 21(IBM Corp, Armonk, NY, USA) data management software. Chi-Square tests were used to measure significance of association or differences between two variables or groups. Score for institutions' entrepreneurship capacity was constructed by summing up 9 items which assessed institution's resources for IPM, ability for commercialisation, involvement of external community partners, support for innovation and creation of new business and entrepreneurship, linkage or engagement with industry and entrepreneurial climate. The generated variable was categorized into comprehensive entrepreneurship capacity and limited entrepreneurship capacity.

Institution's capacity to exploit generated IP was assessed by 5 items which included availability of the following: resources for creation of spin off company, government or regional fund to support IP commercialization, budget for IP protection, licences for ongoing use of digital publications or digital databases, and institution's accessibility to relevant physical and digital information via networking/partnership. After summation of the 5 items, the resulted variable was categorized into comprehensive capacity for IP exploitation and limited capacity for IP exploitation.

Score for institution's IPM capacity was constructed using 7 items which included availability of resources for legal support, fund for operationalization of IP management office (IPMO), unit responsible for evaluating invention's economic prospects and deciding whether to protect and commercialize IP, staff with skills for evaluation of economic prospectus of the invention, staff with business skills, and that institution's strategy align with commercialization goal. The final score was categorized into comprehensive IPM and limited IPM.

Readiness for IPM score was generated by summation of 3 items: scope and volume of research results justify establishment of IPMO, institution's consideration to pool resources with other institutions to manage IP, and reported institution's ability to set up IPMO. For analysis purposes, the score was categorised into comprehensive IPM readiness and limited IPM readiness.

Individual IP capacity was assessed by 6 items which included respondent knowing his/her role in protection and commercialization of IP and where to get IP information, respondent's capacity for protection and commercialization institution's IP, exposure to IP training in the past 5 years, involvement in developing IP policy and entrepreneurship skills. The final individual IP capacity score was constructed by summation of all 6 items and then categorized into comprehensive capacity and limited capacity.

Study participants were skewed towards 2 major institutions of health and allied sciences in Tanzania, and therefore decided to group participants into 3 categories which included 46 participants from National Institute for Medical Research (NIMR), 46 participants from Muhimbili University of Health and Allied Sciences (MUHAS) and 56 respondents from 16 other institutions.

### Ethical Consideration

Data used in this paper were collected through needs assessment study aimed at generating information to inform IP policy developing process for universities and research institutions. The study was granted ethical approval waiver (Ref number NIMR/HQ/R.8a/Vol II of 2020/122) from Medical Research Coordination Committee (MRCC).

## RESULTS

### Respondents' Profile

A total of 148 individuals from health research institutions and universities in Tanzania responded to the distributed online questionnaire, giving a response rate of 59.4%. The majority of respondents per institution were males (78.3%) from Muhimbili University of Health and Allied Sciences (Table 1). Over 70% of respondents have been working with their current institutions for over 6 years.

**TABLE 1:** Profile of Respondents

	Others n (%)	MUHAS n (%)	NIMR n (%)	ALL n (%)	P value
Sex					<.001
Female	20 (36.7)	10 (21.7)	15 (32.6)	45 (30.4)	
Male	36 (64.3)	36 (78.3)	31 (67.4)	103 (69.6)	
All	56 (37.8)	46 (31.1)	46 (31.1)	148 (100.0)	
Age group					<.001
29 - 41 years	27 (48.2)	31 (70.5)	10 (21.7)	68 (46.6)	
42 - 72 years	29 (51.8)	15 (29.5)	36 (78.3)	80 (53.4)	
All	56 (37.8)	46 (31.1)	46 (31.1)	148 (100.0)	

### Perceived Drivers of Institutions' Mission, Priority and Orientation

Most respondents (76.5%) were of the view that social and economic development werethe priorities of their institutions but IP commercialization (29.4%) including databases and software, and meeting local industrial needs didnot form part of the institutions' missions (Table 2). These findings are supported by lack of institutions' strategic direction for IP regulation as only 18% of the respondents reported that their institutions had arrangement with local industries. Furthermore, only 22% of the respondents said that their institutions had functioning IPMOs (Table 3).

**TABLE 2:** Respondents' Perception of Drivers of Institutions' Mission, Priority and Orientation (N=148)

Drivers of institutions' mission	n	%
Academic	96	64.7
Research	113	76.5
IP commercialization	44	29.4
Academic and research	113	76.5
Research and commercialization	44	29.4
Academic, research & commercialization	26	17.6
Institutions' priority		
Humanitarian & philanthropic	61	41.2
Social & economic development	113	76.5
Institution orientation		
Society needs	113	76.5
Academic needs	122	82.4
Local industry needs	44	29.4
Developing & use of databases	87	58.8
Commercialization of databases	44	29.4
Commercialization of software	44	29.4

**TABLE 3:** Institution's Strategic Direction for IP Regulation (N=148)

	n	%
Regulate precise type of IP	55	37.0
Have arrangement with commodity group or industry	27	18.5
Have access to research infrastructure	71	48.1
Have functioning IPMO	33	22.2
Employee responsible for research records and laboratory books	71	44.4

### Awareness on Intellectual Property Management Policies and Related Agreements

**FIGURE 1:**
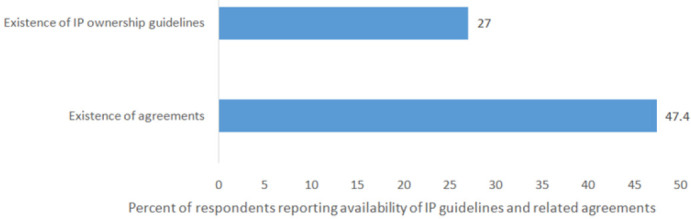
Awareness on Existence of Institutions's Tools For Intellectual Property Management

**FIGURE 2:**
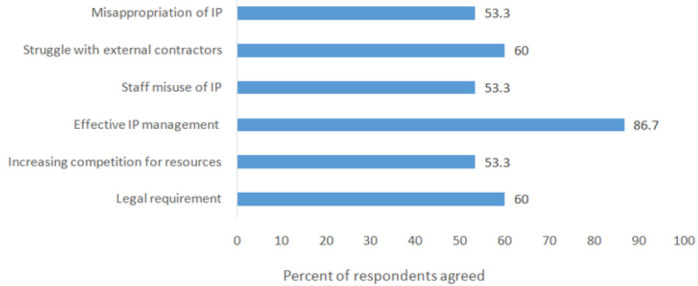
Respondents' Perceived Reasons for Institution to Have Intellectual Property Policy in Place

As shown in Figure 1 and Table 4, approximately 30% of the respondents reported that IP policies existed in their institutions. The frequently mentioned main reason (86.7%) for the institutions to have IP policies in place is to ensureeffective management of created IP (Figure 2). Among respondents who asserted that their institutions had IP policy, 72% reported to have signed employment contract (data not shown in Table or Figure). Over one third to a half acknowledged that their institutions' IP policies intended to regulate mechanisms for benefit sharing and IP ownership (Table 4). Few respondents were aware of the existence of institutions' IP related agreements such as licensing (31.8%) and technology transfer agreement (29.7%).

**TABLE 4:** Presence of Institutional Intellectual Property Policy and regulations (N=148)

	Others n (%)	MUHAS n (%)	NIMR n (%)	ALL n (%)	P Value
Existence of IP policy	21 (37.5)	10 (21.7)	13 (28.3)	44 (29.7)	.001
IP ownership regulation	37 (66.1)	29 (63.0)	23 (50.0)	89 (60.1)	.201
IP use regulation	33 (58.9)	30 (65.2)	23 (50.0)	86 (58.1)	.122
IP commercialization regulation	29 (51.8)	24 (52.2)	19 (41.3)	72 (48.6)	.451
Benefit sharing mechanisms	34 (60.7)	21 (45.7)	17 (37.0)	72 (48.6)	.017
Default legal regime for employee's invention	26 (46.4)	17 (37.0)	8 (17.0)	51 (34.5)	.004
IP ownership of publicly sponsored research regulation	38 (67.9)	25 (54.3)	22 (47.8)	85 (57.4)	.005
IP ownership of privately sponsored research regulation	26 (45.4)	20 (43.5)	21 (45.7)	67 (45.3)	.634
Student's or visiting researcher's IP ownership regulation	27 (48.2)	23 (50.0)	15 (32.6)	65 (43.9)	.462
IP policy implementation	21 (37.5)	15 (32.6)	11 (23.9)	47 (31.7)	.004
Research collaboration policy	52 (92.9)	36 (78.3)	35 (76.1)	123 (83.1)	.534

### Dissemination and Utilization of IP Management Policies and Related Documents

**FIGURE 3:**
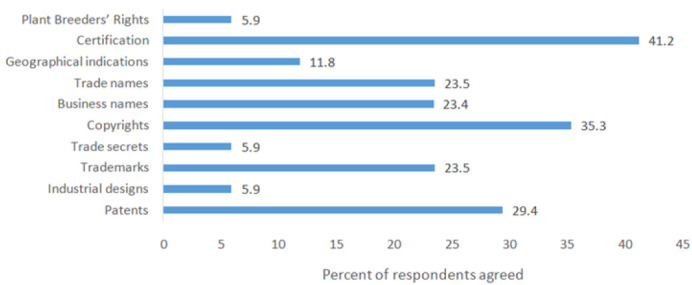
Types of Intellectual Property Rights Granted to the Respondents' Institutions

**FIGURE 4:**
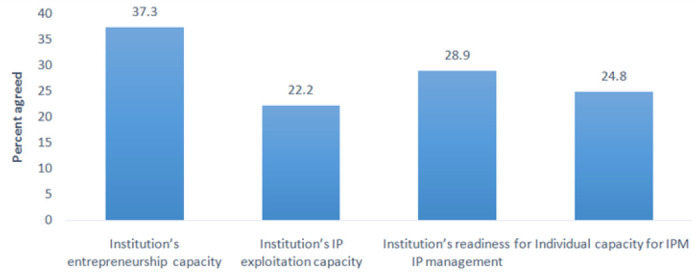
Institutions' Intellectual Property Commercialisation Strategies

Among those who reported that their institutions had IP policies, only 12.8% affirmed that the policies were being implemented: 13.3% (MUHAS), 18.2% (NIMR), and 9.5% other institutions. The majority of respondents (80.4%) knew the existence of employment contracts, but only 28.4% had signed the contract, and 12.8% were well informed that they had been employed to invent (Table 5). According to respondents, the form of IPRs most granted to their institutions included certification, copyrights and patents (Figure 3), and that commercialization was through establishment of joint ventures, exclusive licensing and assignment (Figure 4).

**TABLE 5:** Existence of IP Related Contracts and Agreements (N=148)

	Others n (%)	MUHAS n (%)	NIMR n (%)	ALL n (%)	P Value
Licensing agreement	16 (28.6)	22 (47.8)	9 (19.0)	49 (31.8)	.005
Technology transfer agreement	20 (35.7)	6 (19.6)	15 (32.6)	44 (29.7)	<.001
Employment contract	46 (82.1)	31 (67.4)	42 (91.3)	119 (80.4)	.046
Staff employed to invent	5 (8.9)	6 (13.0)	6 (17.4)	19 (12.8)	.014
Signed employment contract	17 (30.4)	10 (21.7)	15 (32.6)	42 (28.4)	.761

### Capacity and Environment for Intellectual Property Management in Universities and Health Research Institutions

**FIGURE 5:**
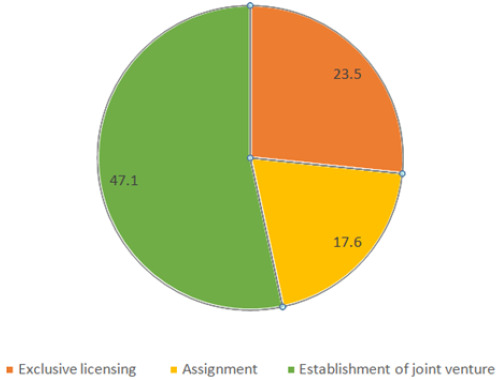
Opinions of Respondents on Universities' and Research Institutions' Capacity for Intellectual Property Management and Entrepreneurship

Respondents were of the opinions that universities and health research institutions had inadequate capacity for IP management as only over 20% of respondents said that their institutions had the capacity to exploit and manage IP, and only a quarter of respondents reported to have capacity for IP management (Figure 5).

In addition, respondents indicated that their institutions do not have conducive environment for IPM. For instance, less than 40% of respondents said that their institutions had entrepreneurship capacity and 30% affirmed that their institutions were capable of establishing IPMO (Figure 5). Only 5.9% of respondents had ever been incentivized for creation of IP and among those, 67.7% expressed dissatisfaction with the incentives provided by their institutions following commercialization of IP they created.

## DISCUSSION

IP policies and related regulations, guidelines and agreements are usually designed to consider the ownership rights, profit sharing and other related rights.^17^ These policies, regulations, agreements and guidelines are important to establish proper management of IPRs, safeguard the process of IP and to provide framework to incentivize researchers in order to promote productivity.^18^ Views of the respondents indicated that there are either gaps in the IP policies of the universities and research institutions in Tanania or respondents have limited knowledge on the contents of their institutional IP policies. These explanations regarding respondents' views are in line with the gaps which were identified in the review of IP policies in academia and research institutions in Tanzania.^19^

Universities and research institutions are perceived as an engine for economic growth through commercialisation of IP.^20^However, environments for accelerating protection and commercialization of research products are not conducive in most academic and research communities in Tanzania. Commercialization is the process of turning a new idea into a marketable product or service^21^ and depends to a large extent on the availability of enabling legislative and policy frameworks that support the effective identification, protection, and management of any intellectual property associated with the R&D results. Commercialisation activities are more valued, if incentives and rewards are provided to researchers in academia and research institutions.^19^

Licensing involve an agreement by the owner of a patent *(licensor)* to allow another party *(licensee)* to make, sell and use the patented invention on an exclusive or non-exclusive basis, without transferring ownership of the patent; hence licensing can be used to generate revenue.^12^ Respondents in the current study indicated that their universities and research institutions rarely use this type of commercialization strategy. Respondents' awareness on existence of institutional IP policies and related regulations, guidelines and agreements is low, even in institutions which have such documents. Lack of dissemination of such information may have contributed to the observed low awareness. Different means of communication channels such as print media, bulletins, internet, videos and WhatsApp should be adopted by universities and research institutions if the goal of raising IP awareness is to be fullfilled. Offering IP awareness and instill a culture of IPM among staff of universities and research institutions. It should be noted that awareness by itself is of little use if institutions do not create and provide suitable systems to enable research communities to protect their rights.^14^ Therefore, it is of little value to raise scientists' awareness on the importance of novelty for getting a patent without supplying them with adequate tools to determine if their inventions are novel or not.

Majority of the respondents were of the views that universities and research institutions in Tanzania lack clear IP policies, regulations, guidelines and agreements that provide guidance on IP ownership, benefit sharing and commercialization. Incentives are rarely provided and in most cases those who received the incentives were dissatisfied. The findings conform with the view that provisions of incentives and rewards for innovators or inventors in academia and research institutions are not systematically organised.^23^

Study findings revealed that respondents perceived their knowledge and skills for IPM are inadequate. Respondents' opinions also indicated that commercialization of IP generated in academia and research institutions in Tanzania is low. The respondents were of the views that negligible proportion of created IP is protected and commercialised. The findings of this study are in line with what have been reported in a study which assessed implementation of IP policy in universities and research institutions in Tanzania.^18^ However, creation of value from IP commercialisation depends very much on what happens before the product is developed^24^ and collaboration between universities, research centres and other organisations.^22^ From respondents' perspective, universities and research institutions in Tanzania have inadequate resources and weak or lack of linkages with industries which may hinder effective commercialization of IP as demonstrated by other studies.^25,26^

Despite the fact that research management centre or technology transfer office plays an important role in developing, coordinating and facilitating commercialisation of IP,^27,28^ respondents in the current study were of the views that their institutions are not well prepared to establish IPMO due to lack of resources and expertise in the relevant field. For universities and research institutions to sustain commercialization of IP, it is crucial to increase IP knowledge and skills among academia and research communities, and improve IPM by nurturing healthy relationship with business partners and facilities.^29^

The ability to leverage IP may require specialised business and/or industry knowledge. Hence, to make the most of the institution's IP holdings, prior knowledge and skills on industry and IPM is required. The industry context and institutional setting matter when it comes to how IP is constructed, used, and deployed. Universities and research institutions in Tanzania are faced with the challenge of realising how knowledge generated through their research base can best be utilized as an asset that can provide maximum value to society, economic and the institution.

### Limitation

Response rate was low, and individuals who responded to the questionnaire may have not been in a position to know the details of the institutions' strategic plans and direction for managing IP and therefore their views may not necessarily reflect the institutions'. Nevertheless, the results of the in-depth interviews and reviews of IP policy documents from various institutions^18^ supported the findings of the online survey in terms of low IP awareness, lack or inadequate implementation of IP policy and limited capacity to manage IP.

## CONCLUSION

Perceptions and views of researchers indicate that universities and health research institutions in Tanzania have inadequate capacity for IPM. According to the respondents, universities and research institutions in Tanzania do not have mechanisms, structures, frameworks and human resource with skills for effective management of IP. Interventions are required for improving institutions' and individuals' capacity to manage IP in Tanzanian health research institutions and universities. In order to create the best environment for IP to be produced and transferred to practical use, universities and research institutions in Tanzania must have a suite of IP policies and practices that reflect their missions, and at the very least ensure that there are arrangements for sharing benefits arising from commercialisation of IP. However, different institutions may put a difference emphasis on the voices of students, research, academic and administrative communities in their policies. Once IP policies are developed, they should be effectively communicated both inside and outside the institution.
